# Computational Modelling of Large Scale Phage Production Using a Two-Stage Batch Process

**DOI:** 10.3390/ph11020031

**Published:** 2018-04-08

**Authors:** Konrad Krysiak-Baltyn, Gregory J. O. Martin, Sally L. Gras

**Affiliations:** 1 Department of Chemical Engineering, The University of Melbourne, Parkville 3010, Australia; konrad.krysiak@unimelb.edu.au (K.K.-B.); gjmartin@unimelb.edu.au (G.J.O.M.); 2 The Bio21 Institute, 30 Flemington Rd, The University of Melbourne, Parkville 3052, Australia

**Keywords:** phage production, modelling, population dynamics

## Abstract

Cost effective and scalable methods for phage production are required to meet an increasing demand for phage, as an alternative to antibiotics. Computational models can assist the optimization of such production processes. A model is developed here that can simulate the dynamics of phage population growth and production in a two-stage, self-cycling process. The model incorporates variable infection parameters as a function of bacterial growth rate and employs ordinary differential equations, allowing application to a setup with multiple reactors. The model provides simple cost estimates as a function of key operational parameters including substrate concentration, feed volume and cycling times. For the phage and bacteria pairing examined, costs and productivity varied by three orders of magnitude, with the lowest cost found to be most sensitive to the influent substrate concentration and low level setting in the first vessel. An example case study of phage production is also presented, showing how parameter values affect the production costs and estimating production times. The approach presented is flexible and can be used to optimize phage production at laboratory or factory scale by minimizing costs or maximizing productivity.

## 1. Introduction

The application of phages in areas such as medicine and food science is a viable alternative to antibiotics that can potentially avoid the problem of multi-drug resistant bacteria. This treatment strategy has received increased attention during the past decade and currently, phages are applied in medicine to treat bacterial infections and in food processing to prevent the contamination of meats [1,2]. Other areas that are actively being explored include the prevention of pipeline corrosion in fracking and the prevention of unwanted foaming in wastewater treatment [3,4]. The scale of these latter applications may require high quantities of phages if the infection rate and phage reproduction rate are low, which is often the case in an environmental setting where nutrient levels are limited [5,6]. The greater popularity of phages and the broader exploration of their potential use is likely to increase global demand. Consequently, we anticipate an increased need for large scale phage production facilities. Such facilities will have a commercial interest in process and cost optimization.

The efficient optimization of phage production processes requires some computational modelling, including models of the population dynamics of bacteria and phages, as these dynamics are non-linear and non-intuitive. Computational models of bacteria and phages have been studied since the late 60’s, with an increasing number of studies appearing in the last decade [7]. Despite this increase in popularity, there do not appear to be any prior studies on computational models in the context of large scale phage production and the optimization of these production processes. It would therefore appear that model development may be lagging behind experimental development in this research area.

In this short communication, we introduce some useful concepts for modelling phage production and develop a computational model that can be used to assist optimization of phage production processes. While the experimental setup selected is simple, the model we present can be applied and extended to other operational conditions where phages and bacteria co-exist, such as production processes in a batch culture in a single bioreactor or production in semi-continuous or continuous chemostat processes.

**Figure 1:**
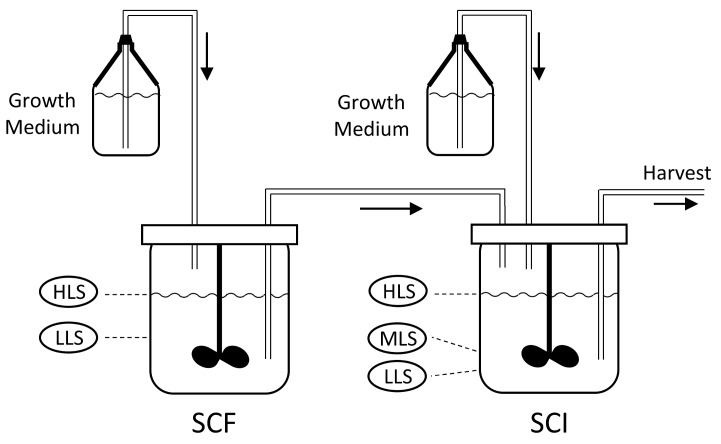
Schematic representation of the two-stage, self-cycling process (see details in text). SCF = the self-cycling fermentation reactor, SCI = self-cycling infection reactor, HLS = high level sensor, MLS = mid-level sensor, LLS = low level sensor.

The goal of this study is to create a model that is simple, whilst retaining the relevant complexity to enable an accurate model of the behavior of phage and bacterial population dynamics. A two-stage, self-cycling process was selected for large scale phage production [8], as this well-known process is suitable for semi-continuous production on a laboratory or industrial scale and can be fully automated. The set-up, shown in Figure 1, consists of two reactors; the self-cycling fermentation (SCF) and self-cycling infection (SCI). Each reactor is supplied with sterile growth medium. 

**Figure 2:**
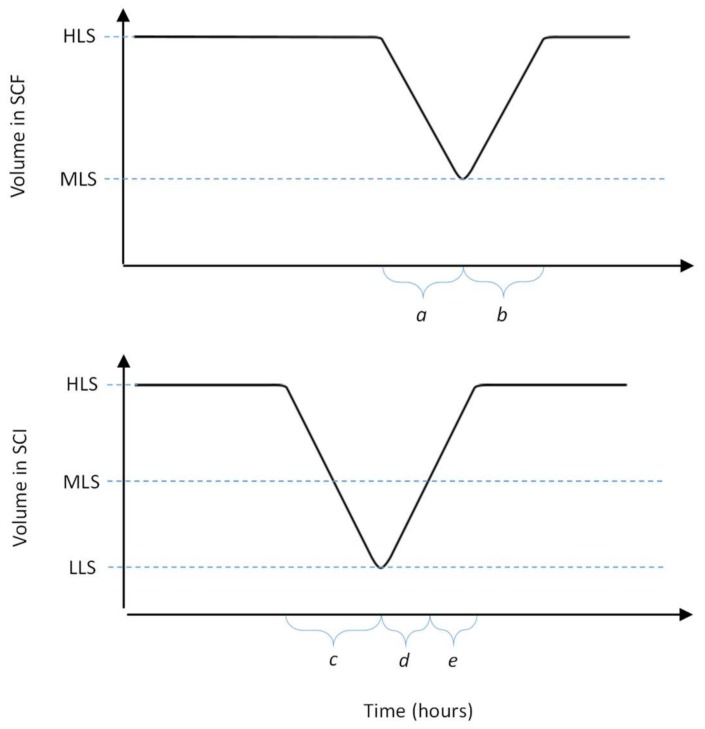
Volume changes in the SCF and SCI reactors during operation, where the volume changes are due to cycling events. Each event involves removal of liquid and subsequent refilling of liquid. (a) transfer of bacterial culture from SCF to SCI; (b) refilling of fresh growth medium into the SCF; (c) removal and harvesting of phages from SCI; (d) the addition of bacteria from SCF until the liquid level reaches the MLS and (e) the addition of fresh growth medium into SCI until liquid level reaches HLS.

The process starts with growing a pure batch culture of bacteria in the SCF reactor, while the SCI reactor is filled to the low level sensor (LLS) with a solution of phages. At regular intervals, a cycling event takes place, which involves the transfer of the liquid medium between the reactors. First, medium is removed from the SCF reactor, such that the liquid level decreases from the high level sensor (HLS) to the LLS. The removed liquid is transferred into the SCI reactor, such that the level within this reactor increases from the LLS to the mid-level sensor (MLS), whilst excess liquid from the SCF is discarded. At this point, fresh growth medium is added to the SCI until the level reaches the HLS. As the SCI contains viable phages, the newly added bacteria get infected, which leads to lysis and the release of new phage progeny. In parallel, fresh growth medium is added to the SCF until the level reaches the HLS, starting a new cycle of bacterial growth. After a period of phage production, the contents of the SCI reactor are removed until the LLS is reached. The removed liquid is then subjected to downstream processing and harvesting steps to collect phages. Between the cycling events, the SCF and SCI reactors act as batch processes. The changes in the levels of growth medium in each of these stages is shown in Figure 2.

Evolution of the species of bacteria and phages used can significantly alter the production process if not addressed properly. Co-evolution between bacteria and phages occurs readily when they are grown together in continuous cultures such as a chemostat [9,10,11]. In particular development of bacterial resistance against phages could lead to significantly lower phage productivity, making continuous cultures unsuitable for large scale production. The two-stage, self-cycling process employed here is a type of modified batch process, where bacteria are first grown separately from phages and the phages are only introduced once the bacterial population has reached an appropriate size. Bacterial resistance to phages is therefore not favoured during the growth of bacteria in the SCF and is less likely to occur in the SCI reactor for several reasons; (a) as the concentration of bacteria is high, the rate of infection will also be high and lead to a swift eradication of bacteria; (b) the batch design will prevent any resistant bacteria, which will have a low initial concentration, from growing over an extended period of time. 

The two-stage, self-cycling process has been confirmed experimentally to successfully operate without showing evidence of resistant bacteria or more virulent phages developing [8], and the evolution of resistance can therefore be ignored in the computational model developed here. It must be noted, however, that co-evolution can only be prevented if the phages in the SCI reactor are prevented from contaminating the SCF reactor; thus careful design is crucial to ensure unidirectional flow. 

General evolution due to random mutational changes can slowly alter the characteristics of the bacteria and phages over longer periods of time [12] and may be unavoidable in a longer term industrial process. This process must be addressed through quality control by inspecting productivity and testing the infectivity of the produced phages on fresh bacterial cultures grown from frozen stock. If deviation in the phage infectivity is detected, the production process must be stopped, the reactors cleaned and sanitized and the production process restarted with fresh cultures.

An appropriate set of assumptions and biological mechanisms must be included to formulate a suitable computational model that can simulate phage production. Traditionally, models of phages and bacteria have been formulated using *delay differential equations* (DDEs), described in detail in the *materials and methods* section. When more than one reactor is included, as in the case considered in Figure 1, DDEs are problematic and it is therefore advisable to reformulate the basic models (Equations (5)–(8)) into a system of *ordinary differential equations* (ODEs). This process has been described in detail previously [13]. Reformulating the DDEs into ODEs requires that the infected bacteria $X_{I}$ is subdivided into N subpopulations ($X_{I,1}$, $X_{I,2},$…, $X_{I,N}$), where each subpopulation represents a certain “age” after infection. In this notation, $X_{I,1}$ would be the youngest bacteria immediately after infection, while $X_{I,N}$ would be the oldest population immediately before lysis. The idea is that the bacterial populations $X_{I,1}$ undergoes ageing by being “transferred” into population $X_{I,2}$ and so on. With this approach, no delay terms (T−t) are necessary and a higher value of N would in general lead to a better approximation of the DDE model. 

While the batch process allows us to ignore bacterial resistance, this set up causes another complication, as the phage infection rate varies as a function of bacteria-specific growth rate μ (or indirectly substrate availability) and this variation must be considered within the model. In many models published to date, the infection parameters of $K_{i}$, T and b are assumed constant throughout the simulation [9,10,14,15,16,17]. This assumption may be appropriate if simulating a continuous culture, where the substrate concentration remains fairly constant. Phage infection parameters have, however, been shown experimentally to vary considerably as a function of specific growth rate. Specifically, the adsorption rate has been observed to vary 1–2 orders of magnitude, burst size to vary from 0–150 depending on the species and the latency period to exhibit at least a two-fold difference in magnitude [5,6,18,19].

If the substrate concentration changes significantly to relatively low levels during the course of simulation, as could be expected in a batch process, variable infection parameters should be implemented, as past work indicates it will improve model accuracy [20]. This may also be expected in the phage production processes, described in Figure 1, where the substrate is unusually expensive or where the substrate concentration cannot easily be controlled. An example of the latter is phage production applied under the same conditions that are present during fracking, which can help eradicate bacteria that can contribute to pipeline corrosion [21].

## 2. Results

To illustrate the potential improvement in accuracy with a model that incorporates variable infection parameters, we performed simulations of a batch process using the basic model described in Equations (5)–(8) and the proposed improved model described in Equations (13)–(19) (Supplementary Figure S1). These simulations were then compared to experimental data previously reported in the literature [20]. As the previous study determined infection parameters only during the bacterial log growth phase and no further data on the variability of infection parameters exists as a function of growth rate for the particular bacteria-phage pairing under consideration, we tested models where the variable infection parameters were varied over a range that was based on the study by Santos et al. and generally reported values in other past studies. The proposed model was able to produce a much better fit to the experimental data using the variable infection parameters selected from the appropriate range; an observation that can be more thoroughly tested when a wider range of experimentally determined parameters are available. 

To demonstrate the practical application of the proposed model presented here, we simulated the two-stage, self-cycling process for phage production at a 50 L scale, which reflects industry practice. The parameters within the biological model were chosen based on past experimental studies from the literature [5,6,17,18]. Parameters relating to bacteria growth were $\mu_{max}=0.7726$ /h, $K_{m}=0.0727$ µg/mL and $e=10^{-6}$ µg/cell. Parameters describing the infection rate were varied as a function of the specific bacterial growth rate μ. 

The infection rate $K_{i}\left(\mu \right)$ was assumed to vary exponentially as a function of the specific growth rate from $K_{i}\left(0\right)=10^{-9}$ to $K_{i}\left(\mu_{max}\right)=10^{-7}$, summarized in Equation (1):

(1) $$K_{i}\left(\mu \right)=10^{-9+2\cdot \frac{\mu }{\mu_{max}}}\;\mathrm{for}\;0\leq \mu \leq \mu_{max}$$

The latency time T was varied linearly from 0.8 h down to 0.4 h whilst burst size b was varied linearly from 0–100 phages/cell with increasing μ:

(2) $$T\left(\mu \right)=0.8-0.4\frac{\mu }{\mu_{max}}\;\mathrm{for}\;0\leq \mu \leq \mu_{max}$$

(3) $$b\left(\mu \right)=90\frac{\mu }{\mu_{max}}\;\mathrm{for}\;0\leq \mu \leq \mu_{max}$$

In this study, we have defined the functions $K_{i}\left(\mu \right)$, T(μ) and b(μ) in such a way to make the parameter values range between values reported in the literature for *E. coli* [5,6,17,18]. These values, however, will differ if different bacteria or phage strains are used or if conditions of the production process change, such as different pH, growth media or temperature. The infection parameters, and their dependence on bacterial growth rate and potentially other variables, must be estimated for each new species of bacteria and phage as well and experimental conditions.

The model was optimized to minimize production costs, measured as the cost per phage particle, by varying 5 operational parameters within a given range (Table 1), followed by an assessment of parameter interactions for the optimal parameter values (Table 2). We employed a semi exhaustive search-scheme, where 4 different values were tested for each parameter (Table 1). As 5 parameters were varied, this gave rise to $4^{5}=1024$ model simulations. Once an optimal value was found, the same scheme was employed a second time but within a narrower range (equal to 1/3 of the initial range) to fine tune the optimal value. A total of 1024×2=2048 simulations were performed.

**Figure 3:**
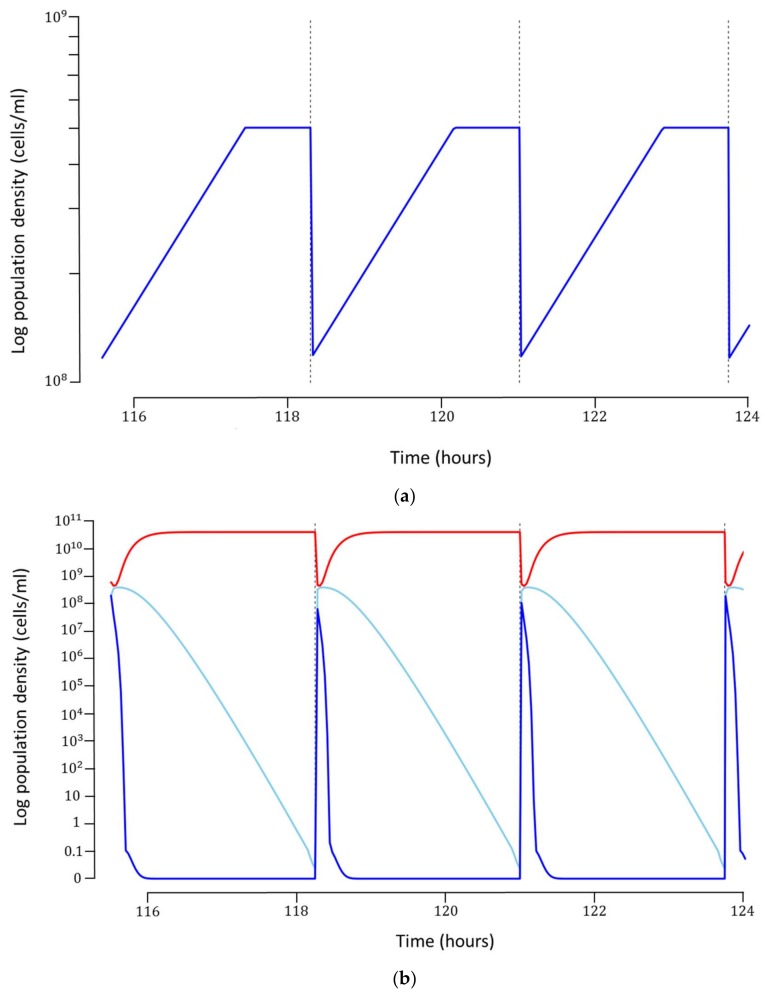
The concentration of bacteria and phages during simulation of the two-stage, self-cycling process for phage production. (**a**) SCF-tank where only susceptible bacteria $X_{S}$ (blue line ▅) are grown; (**b**) SCI tank where susceptible bacteria $X_{S}$ (blue line ▅), infected bacteria $X_{I}$ (turquoise line ▅) and phages P (red line ▅) are grown. Vertical bars represent cycling events, where phages are harvested from the SCI tank and bacteria are transferred from the SCF to SCI tank.

Each simulation was run for 45 cycles, which was long enough to ensure that a steady-state was reached. Once steady-state was obtained, the production cost for each cycle was calculated based on the hourly costs of operation, maintenance and utilities, as well as cost of preparation of growth medium (Table 3). These costs are illustrative and would need to be determined on a case by case basis. It is noteworthy that the optimal model also requires a high concentration of bacteria, ranging between 10^8^–10^9^ cells/mL, to be maintained inside the SCF reactor during operation (Figure 3a). It can be argued that such a concentration of host bacteria may be too high to support robust phage infection. If this is indeed the case for the phage and bacterium under consideration, a host concentration dependent infection rate can be incorporated into the model, provided experimental data can be generated. In our model, we have omitted this potential mechanism.

The optimal model can produce about $1.923\times 10^{15}$ phages/cycle at a concentration of about $4\times 10^{10}$ phages/mL (Figure 3b). The total cost for each phage particle was $$4.41\times 10^{-13}$ (Table 4). In the 2048 simulations performed in the semi-exhaustive search scheme, we observed that the cost per phage particle varied considerably, with some of the most pessimistic model outcomes producing phages at a cost of roughly $$3\times 10^{-10}$ per particle, which is three orders of magnitude more expensive than for the optimal model. Similarly, the productivity, i.e., phages produced per time unit, varied over three orders of magnitude between the best and the worst model outcome. In some scenarios with very unfavorable conditions (such as very low substrate concentration), no phages were produced for the duration of the simulations. Whilst the absolute costs will vary as a function of the operational inputs (Table 3), this data highlights the utility of this approach.

Once optimal parameter values were found with the semi-exhaustive search scheme described above, a local sensitivity analysis was performed independently, one-at-a-time, for each parameter. The sensitivities indicate the parameters that have the greatest influence on production cost. We define sensitivity as the relative change in production cost divided by the relative change in a given parameter value. Mathematically this can be written as:

(4) $$S=\frac{\Delta C/C}{\Delta V/V}$$

where S = sensitivity, C = cost per phage particle, V = any given parameter value.

The operational parameter values are shown in Table 1 together with the sensitivity of the model to each parameter. Interestingly, for the optimal model the substrate concentration in the influent to the SCI reactor was about 69% lower than in the influent to the SCF reactor, indicating that different growth media have to be prepared for each reactor. The substrate concentration S in the SCF influent was found to have the greatest effect on production cost, giving the highest absolute value for sensitivity. The sensitivity of $-9.7\times 10^{-1}$, given in Table 1, indicates that at the point where the parameter values are optimal, a 100% increase in S would lead to a further 97% decrease in production costs. The second most important variable is the LLS in the SCF reactor. The sensitivity of $3.0\times 10^{-1}$ indicates that at the point of optimal parameter values, a 100% decrease in the LLS would decrease production costs by 30%. 

The parameter sensitivities extracted from the computational model can offer guidance for process optimization in a laboratory or on the factory floor by pointing to the parameter with the greatest impact on production outcome. It is important to realize that, due to the highly non-linear nature of the model, interactions between parameters will likely exist. This will mean that the sensitivity of each parameter will differ based on the values of the remaining parameters. Interactions between parameters were investigated locally around the optimal point by slightly perturbing two parameters at a time. The magnitude of the interaction was estimated by performing a large number of simulations after slightly perturbing the parameter values and then performing multiple linear regression on the normalized parameter values and outcome.

An interaction was considered significant if the *p*-value was <0.05 after applying a Bonferroni correction for multiple comparisons (i.e., the original *p*-value was multiplied by 10, as a total of 10 pairwise interactions were tested). Three interactions were found to be statistically significant (Table 2), with the most significant interaction being between the LLS in the SCI tank and the MLS in the SCF tank. Both the LLS in the SCF tank and the cycling time appear twice among the three interactions found. This result indicates that in the vicinity of the optimum parameter values, the outcome of small perturbations to the cycling time or LLS are more likely lead to unpredictable results.

An interesting case study is the application of phages during fracking and this will be considered in greater detail next. Pipeline corrosion starts immediately once fracking water has been added to a well [3]. Problematic bacteria should therefore be neutralized quickly. Assuming a target cell concentration of $10^{6}$ cells/ml (as indicated by [21]), a constant $K_{i}=10^{-7}$ mL/h and a constant latent period of 0.5 h, simulations using the basic model predict that roughly $10^{7}$ phage particles/ml are necessary to eradicate more than 90% of bacteria within an hour (Supplementary Figure S2). As a fracking well can require around 18 million litres of water, a total of $10^{16}$–$10^{17}$ phage particles may need to be dosed. A timeframe of roughly 140 h would be required to produce the required number of phages using the parameter values in the model presented in this study. This time frame is highly dependent on the infection parameters chosen in the model, hence the order of magnitude difference in the estimate. In this study, the values for infection parameters as a function of growth rate were obtained from the literature [5,6,17,18]. 

## 3. Discussion

A computational model suitable for modelling phage production has been formulated by integrating variable infection parameters as a function of bacterial growth rate. Previous modelling has suggested that such variable infection parameters can improve model accuracy [20], although the lack of experimental data on variable infection parameters determined for bacteria at different stages of growth prevents direct comparison. Here, the potential for an improvement in accuracy of the proposed model (Equations (13)–(19)) was demonstrated by comparing the simulated output to the output generated by the basic model (Equations (5)–(8)) and experimental data obtained from [20] by allowing the infection parameters to vary across the range reported in past studies in the literature. Although the proposed model has a better fit to experimental data, this comparison should not be understood as a proof that the more complex model is superior but rather an indication that there is potential for this model, which contains more parameters, to be more accurate. The future experimental determination of infection parameters over a range of different growth rates, ideally also from batch cultures grown under a number of different conditions will enable the model presented here to be more thoroughly evaluated.

The practical application of the proposed model was showcased by incorporating the proposed bacteria-phage dynamics into a simulation of the two-stage, self-cycling process. The main practical application of modelling the two-stage, self-cycling process is the ability to find optimal process conditions, i.e., values of operational parameters such as sensor levels, cycling times and substrate concentrations that produce optimal outcomes. 

In commercial applications, a common objective is to minimize the cost of production, defined here as cost per phage. In small scale phage production, we would expect that the operational costs (including salaries for operators) would dominate whilst the cost of bacterial growth medium would play a smaller role. The opposite is expected in large scale production [22]. For the example illustrated here, the substrate concentration in the SCF influent most influenced production costs, followed by the low level setting in the SCF reactor. Both the production costs and productivity varied by roughly three orders of magnitude between the worst and the best model outcomes, with some models producing no phages at all under very unfavorable conditions. 

The great variability in model outcomes illustrates the usefulness of modelling as a means to assist in the phage production process optimization, especially as the system under consideration is highly non-linear and difficult to predict intuitively. Due to the non-linear nature of the system, it is likely that other local minima in production costs could be found using other sets of parameter values that differ significantly from the values presented in Table 1. Whether those minima are more preferable or not will likely depend on the constraints imposed by the production process under consideration. 

Another factor to consider is the “robustness” of any local minimum. There is a potential challenge in replicating the simulated conditions in real-world experimental set-ups, as the model is not necessarily completely accurate and as the experimental measurements contain errors and potentially other sources of variation. Using the parameter values presented in Table 1 could therefore lead to a slightly different outcome in practice. To take this into account, the optimal parameter values can be chosen based on both the outcome and the likelihood of successfully replicating those conditions experimentally. In this regard, it may be preferable to find a local minimum that does not vary much in the surrounding parameter space. If production costs stay close to the local minimum in a large “area” of the local parameter space, the conditions are more likely to be successfully replicated experimentally. 

To ensure model accuracy, the biological system under consideration must be well characterized by measuring the relevant biological parameters including growth and infection parameters. This is an area where further experimental work could improve our understanding of phage dynamics and lead to greater model accuracy by establishing the relationship between infection parameters and bacterial growth rate. While there has been a lack of published computational models describing phage production, models of production of virus-like particles (VLPs) in insect cells with recombinant technologies have been extensively studied [23,24]. 

VLPs produced in insect cells are often included in vaccines, as they can present antigens to the immune system but are not actively infectious and pathogenic [23]. The process of VLP production can be quite complex, requiring the use of expression vectors with multiple genes for producing components of the complex viral capsid, chaperones and enzymes involved in post-translational modification. As such, the major challenges have been to produce expression vectors that could successfully produce intact and complete VLPs. Consequently, much of the modelling effort in this area has focused on intracellular processes and the prediction of the number of intact VLPs as a function of culture conditions [24,25]. Whilst models for VLP production that include population dynamics have been described [26,27], the authors were unable to find any such models that include the influence of substrate availability on infection parameters, as well as the engineering parameters that directly affect unit operations, as was examined here.

The modelling approach described here could be useful to guide phage as well as VLP production on a laboratory or industrial scale, pinpointing the operational parameters that have the greatest influence on cost or productivity. This model can also be adapted to optimize other properties of interest including the production time, yield or final product concentration. One of the most important features of the model described in this study is the flexibility of the approach; as the biological interactions have been reformulated into a set of ODEs, they can be implemented into models containing an arbitrary number of interconnected reactors. The approach taken can therefore be applied to any other scenario where bacteria and phage interactions are expected at a laboratory or manufacturing scale or during the application of phages, provided the infection parameters for the phages and bacteria can be experimentally characterized [28].

## 4. Conclusions

A model has been proposed describing phage-bacteria population dynamics that incorporates variable infection parameters as a function of growth rate. The model has furthermore been formulated as a set of ODEs, to allow the flexibility to simulate multi-reactor systems consisting of any arbitrary number of interconnected reactors. Model simulations of batch processes indicate that the proposed model may potentially be more accurate than the basic model of bacteria-phage dynamics, although further work is required to confirm this prediction. Incorporating the model into a two-stage, self-cycling process allows for phage production costs to be minimized *in silico*, avoiding the need for costly experiments to be run under different conditions. Our simulations indicate that costs can vary several orders of magnitude depending on the process conditions, demonstrating that the proposed computational model could prove useful for the optimization of phage production.

## 5. Materials and Methods 

The basic computational models of bacteria-phage population dynamics that must be included in models for phage production take into account four processes: bacterial growth, bacterial decay, phage decay and the infection of bacteria by phages. These models are most commonly formulated into systems of DDEs. An example of a fairly simple model consisting of one species of bacteria, one phage and one substrate grown in a single batch reactor where the volume is kept constant over the course of time is given by Equations (5)–(8):

(5) $$\frac{dS}{dt}=-e\;X_{S}\;\mu \left(S\right)$$

(6) $$\frac{dX_{S}}{dt}=X_{S}\mu \left(S\right)-K_{i}X_{S}P-d_{X}X_{S}$$

(7) $$\frac{dX_{I}}{dt}=K_{i}X_{S}P-e^{-DT}K_{i}X_{S}\left(t-T\right)P\left(t-T\right)-d_{X}X_{I}$$

(8) $$\frac{dP}{dt}=be^{-d_{X}\cdot T}\;K_{i}X_{S}\left(t-T\right)P\left(t-T\right)-K_{i}X_{S}P-d_{P}P$$

where:

S = substrate concentration (µg/mL)
$X_{S}$ = concentration of susceptible bacteria (mL^−1^)
P = concentration of phages (mL^−1^)
μ(S) = bacterial specific growth rate (s^−^^1^) as a function of S
b = burst size (phages/cell)
$X_{I}$ = concentration of infected bacteria (mL^−1^)
$K_{i}$ = adsorption rate constant (mL/h)
T = latent period (h)
$d_{P}$ = decay rate of phages (h^−^^1^)
$d_{X}$ = endogenous decay rate of bacteria (h^−^^1^)
e = substrate required to produce a bacterial cell (µg)

The Monod growth expression is most commonly used to model the bacterial specific growth rate μ given by:

(9) $$\mu \left(S\right)=\frac{\mu_{max}\;S}{K_{m}+S}$$

where:

$\mu_{max}$ = the maximum bacterial specific growth rate (s^−1^)
$K_{m}$ = half saturation constant

Three parameters characterize the mechanism of phage infection and lysis, including the adsorption rate $K_{i}$, the latent period T and the burst size b. The rate at which phages adsorb to a bacterial cell surface is given by $K_{i}\;X_{S}\;P$, assuming mass action kinetics. Once adsorbed, a phage injects its genome into the bacterial cell. If the phage is in the lytic cycle, then new phages are produced until the bacterial cell lyses. The time from adsorption to lysis is termed the latent period T. The number of phage particles released from a single cell upon lysis is termed the burst size b. The rate at which new phages are produced is given by the expression $\;b\cdot e^{-d_{X}\cdot T}\;K_{i}X_{S}\left(t-T\right)\;P\left(t-T\right)$. The terms $X_{S}\left(t-T\right)$ and P(t−T), commonly referred to as *delay terms*, represent the concentrations of phages and susceptible bacteria at time T prior to the current time point t and form the basis for DDEs. The basic model given by Equations (5)–(8) was first introduced in the 1970’s [10] and still forms the basis for recently published models, which include additional biological mechanisms such as bacterial resistance, multiple host binding sites and other features. We refer the reader to an extensive review on this subject for more details [7].

As stated in the introduction section, DDEs cannot easily be applied to models containing multiple interconnected reactors. To circumvent this problem, the DDEs can be reformulated into ODEs. The basic model given by Equations (5)–(8) can be reformulated into ODEs by subdividing the infected bacteria $X_{I}$ into N subpopulations ($X_{I,1}$, $X_{I,2}$, …, $X_{I,N}$), where each subpopulation represents a certain “age” after infection [13]. In this notation, $X_{I,1}$ would be the youngest bacteria immediately after infection, while $X_{I,N}$ would be the oldest population immediately before lysis. The idea is that the bacterial populations $X_{I,1}$ undergoes ageing by being “transferred” into population $X_{I,2}$ and so on. With this approach, no delay terms (T−t) are necessary and a higher value of N would in general lead to a better approximation of the DDE model.

Implementing variable infection parameters has been done previously with DDEs [20]. However, in the case of multiple interconnected reactors, this functionality must be formulated as a set of ODEs, which can be done by subdividing each infected bacterial population $X_{I}$ into M×N subpopulations, with each subpopulation denoted by $X_{I,m,n}$ where 1≤m≤M and 1≤n≤N. The index of n indicates the age group of the infected population (see [13]). In contrast, the index of m indicates subpopulations with different infection parameters, $K_{i,m}$, $T_{m}$ and $b_{m}$.

At each time point during simulation, the bacterial specific growth rate μ dictates the M infected subpopulation $X_{I,m,1}$ (where 1≤m≤M) that will increase due to phage adsorption and infection. We define a monotonically increasing function σ(μ) that can output any integer between 1 and M. At each point in time, only the population $X_{I,\sigma \left(\mu \right),1}$ will increase in concentration due to phage adsorption at a rate given by $K_{i,\sigma \left(\mu \right)}X_{S}P$. All subpopulations $X_{I,m,1}$ continuously “age” to produce a burst of $b_{m}$ phages per cell after the latent period $T_{m}$. Past studies have indicated that an increase in the growth rate μ is expected to lead to an increase in the infection rate $K_{i}$ and burst size b, whilst a decrease is expected in the latent period T [5,6,18]:

(10) $$K_{i,1}<K_{i,2}<\cdots <K_{i,M}$$

(11) $$b_{1}<b_{2}<\cdots <b_{M}$$

(12) $$T_{1}>T_{2}>\cdots >T_{M}$$

The exact relationship between μ and $K_{i}$, T and b must be measured experimentally and will depend on the experimental conditions and the chosen species of bacteria and phages. Pure cultures of bacteria should be grown at several different dilution rates in chemostats. Once steady-state is reached, a sample should be withdrawn and used to estimate infection parameters according to established methods [29] . The steps involved in these experimental methods have also been outlined recently [13].

Equations (13)–(19) describe a system of ODEs in a single well-mixed reactor that incorporates variable infection parameters as a function of μ. As no delay differential equations are included, this system can readily be applied to multi-reactor models, such as the two-stage, self-cycling process shown in Figure 1:

(13) $$\frac{dS}{dt}=-X_{S}\;\mu \;e$$

(14) $$\frac{dX_{S}}{dt}=X_{S}\;\mu \left(S\right)-X_{S}\;d_{X}-K_{i,\sigma \left(\mu \right)}\;X_{S}\;P$$

Changes in the “young” infected populations where n=1:

(15) $$\frac{dX_{I,\sigma \left(\mu \right),1}}{dt}=K_{i,\sigma \left(\mu \right)}X_{S}\left(t\right)P\left(t\right)-\left(d_{X}+D_{T,\sigma \left(\mu \right)}\right)X_{I,\sigma \left(\mu \right),1}\left(t\right)$$

(16) $$\frac{dX_{I,m,1}}{dt}=-\;\left(d_{X}+D_{T,m}\right)\;X_{I,m,1}\left(t\right)\;\mathrm{for}\;1\leq m\leq M\;\mathrm{and}\;m\neq \;\sigma \left(\mu \right)$$

Changes in the “older” infected populations where n>1:

(17) $$\frac{dX_{I,m,n}}{dt}=D_{T}\;X_{I,m,n-1}-\left(d_{X}+D_{T,m}\right)X_{I,m,n}\left(t\right)\;\mathrm{for}\;2\leq n\leq N\;\mathrm{and}\;1\leq m\leq M$$

and for the phage population:

(18) $$\frac{dP}{dt}=-K_{i,\sigma \left(\mu \right)}X_{S}P+\overset{M}{\underset{m=1}{\sum }}b_{m}\cdot D_{T,m}\;X_{I,m,N}-d_{P}P\left(t\right)$$

and:

(19) $$D_{T,m}=\frac{N}{T_{m}}$$

where:

S = substrate concentration (µg/mL)
$D_{T,m}$ = ageing rate of infected bacteria m (h^−1^)
P = concentration phages
$X_{S}$ = concentration of susceptible bacteria (mL^−^^1^)
b = burst size (phages/cell)
$K_{i,m}$ = adsorption rate constant (mL/h)
T = latent period (h)
e = substrate required to produce a bacterial cell (µg)
$d_{X}$ = decay rate of bacteria (h^−1^)
$d_{P}$ = decay rate of phages (h^−1^)
μ = bacterial specific growth rate as a function of substrate S (h^−1^)
N = number of discrete steps to represent the course of the latent period
M = number of discrete populations to represent $K_{i,m}$, $T_{m}$ and $b_{m}$ as function of μ
$X_{I,m,n}$ = concentration of infected bacteria population m at age n (mL^−^^1^)
σ(μ) = a function specifying which young infected population $X_{I,m,1}$ should increase in concentrationdue to infection, with 1≤σ(μ)≤M.

The phage production models were simulated using a script (developed in-house) written in the R statistical language. The software was executed on a standard laptop by Hewlett Packard with 2.60 GHz Intel i7-6600 CPU, 16 Gb RAM and 64-bit windows 7 operating system.

A semi exhaustive search-scheme was used to find parameter values that minimize production costs. Five parameters were varied, and four different values were tested for each parameter, which amounted to $4^{5}=1024$ model simulations. To fine tune the optimal value, the same scheme was repeated a second time within a narrower search range. A total of 1024×2=2048 simulations were performed, which took a total of 36 min.

A local sensitivity analysis was performed around the point where optimal parameter values were found. This was done by varying each parameter one-at-a-time, a distance of ±1% away from the mid-point. The production costs obtained from these simulations were then compared to the cost obtained at the mid-point to calculate sensitivities. With five parameters, a total of 5×2=10 simulations were performed. 

Interactions between parameters were investigated by perturbing two parameters at the same time around the optimal point and performing a simulation to obtain an outcome. Parameters were perturbed −2%, −1%, 0%, 1% and 2% away from the optimum. With 5 levels and 5 variables, the number of simulations performed was $5^{5}=3125$, which took a total of 54 min. The outcomes and parameter values for each simulation were recorded and the data was then normalized. The normalized data was then analysed by multiple linear regression with all possible pairwise interaction terms included.

## Figures and Tables

**Table 1:** Operational model parameters employed for the simulations.

			**Sensitivities**	
**Optimized Parameters**	**Ranges Tested**	**Value for Optimal Model**	**Cost per Phage**	**Phage Productivity**
LLS (in SCF)	10–40 L	11.6 L	$3.0^{-1}$	$-3.0\times 10^{-1}$
MLS (in SCI)	10–40 L	40.0 L	0	0
Cycling time	0.5–5 h	2.75 h	$1.3\times 10^{-1}$	$-9.9\times 10^{-1}$
Concentration of substrate S in influent for SCF	1–1000 µg/mL	1000 µg/mL	$-9.7\times 10^{-1}$	$9.9\times 10^{-1}$
Concentration of substrate S in influent for SCI	1–1000 µg/mL	316 µg/mL	$3.8\times 10^{-4}$	$1.7\times 10^{-5}$
**Constant Parameters**	**Value**			
HLS (in SCF)	50 L			
HLS (in SCI)	50 L			
LLS (in SCI)	1 L			

**Table 2:** Parameter interactions by determined by multiple linear regression around the optimum parameter values.

Interactions	Estimated Coefficient	Adjusted *p*-Value
-LLS (in SCF) -MLS (in SCI)	14	$13\times 10^{-71}$
-Cycling time -Concentration of substrate S in influent for SCF	52	$26\times 10^{-11}$
-Cycling time -LLS (in SCF)	2.5	$97\times 10^{-3}$

**Table 3:** Costing and operational expenses.

Expenses	Amount ^1^
Operation (including personnel and utilities)	$40/h
Substrate cost	$0.1/g
Liquid medium (including preparation and sterilization)	$15/L

^1^ The prices for substrate and sterilized liquid media were chosen to reflect actual and current prices from common suppliers including Sigma.

**Table 4:** Productivity and production costs for the optimal model.

Cost and Productivity for Optimal Model	
Cost per phage particle	$$4.4\times 10^{-13}$/phage particle
Cost per hour	$309/hour
Cost per cycle	$850/cycle
Phages produced per hour	$7.0\times 10^{14}$ phages/hour
Phages produced per cycle	$1.9\times 10^{15}$ phages/cycle

## Supplementary Materials

The following are available online at http://www.mdpi.com/1424-8247/11/2/31/s1.
