# Supplementary material for: Computational Modelling of Large Scale Phage Production Using a Two-Stage Batch Process
Source: Pharmaceuticals (Basel). 2018 Apr 8;11(2):31. doi: 10.3390/ph11020031 (PMC6026895; doi:10.3390/ph11020031)
Supplement: Supplementary file 1 [file pharmaceuticals-11-00031-s001.pdf]

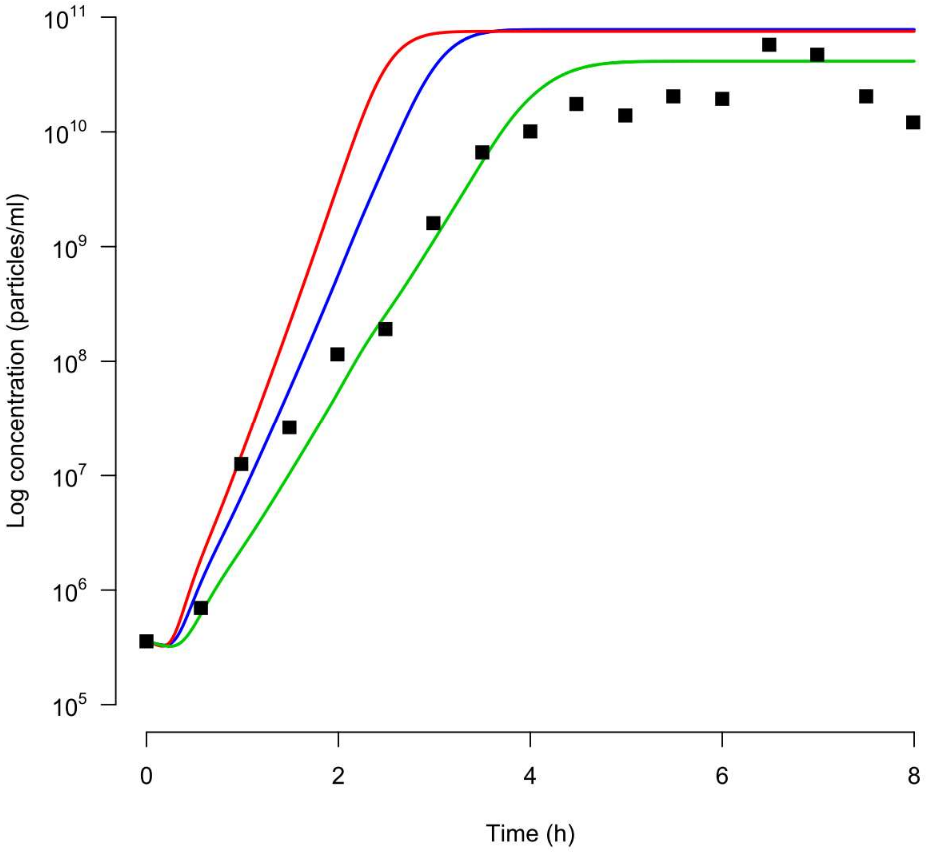

Figure S1. Simulation showing phage concentration over time in models of phage-bacteria interactions in a batch reactor, with one basic model that includes constant infection parameters (red line) and two models that include variable infection parameters (blue and green lines). The simulations are compared to experimental data obtained from (Santos *et al.* 2014) for phage-bacteria interactions showing how phage concentration changes over time (solid black squares).

The basic model is described in equations 5-8 in the main text, while the proposed improved models that include variable infection parameters are described in Equations **Error! Reference source not found.**3-19 in the main text. The basic model used infection parameters estimated experimentally by Santos and colleagues (Santos et al. 2014) for bacteria grown only in the log phase: $K_i = 10^{-9}$ ml/h, $b = 58$, $T = 0.5$ h. Santos *et al* did, however, not experimentally determine the variation of the infection parameters at different growth rates. Due to this lack of data, in the second and third model (blue and green line) model these parameters were varied across a range similar to the variation in these parameters observed in the literature, although for the third model (green line) the parameter values were also manually adjusted to generate a better fit.

The experimentally determined parameter values obtained during the log phase by Santos *et al.* likely correspond to the highest possible infection rate that could take place with the bacteria-phage pairing under consideration, i.e. the maximum adsorption rate and burst size and the lowest latency time. Here, the adsorption rate and burst size were set to decrease with decreasing bacterial growth rate, while the latency period was set to increase with decreasing bacterial growth rate. For the second model (blue line) the parameter values for the adsorption rate were set to $10^{-11} \leq K_i \leq 10^{-9}$ (varied exponentially two orders of magnitude below the maximum rate in the basic model), the latency period to $0.5 \leq T \leq 1.0$ (varied linearly by a factor of 2) and the burst size was set to $0 \leq b \leq 58$ (varied linearly).

The fit between the simulated output and experimental data was reasonably poor for the basic model (red line) and the second model (blue line), although it was slightly better for the second model that incorporates

variable infection parameters than for the basic model. This comparison should only be considered an indication of the potential of the more advanced model to improve accuracy and model prediction, as a comprehensive comparison is limited by the lack of experimental data describing how the infection parameters vary with bacterial growth. We were able to greatly improve the fit for the proposed model by letting the latency period vary from 0.5 – 2h and the burst size vary logarithmically between 0.01 and 58 (green line). This improvement , however, needs to be confirmed experimentally. The proposed model therefore appears to represent an improvement on the basic model, although experimental data on the variation of the infection parameters as a function of bacterial growth rate is required to allow further validation.

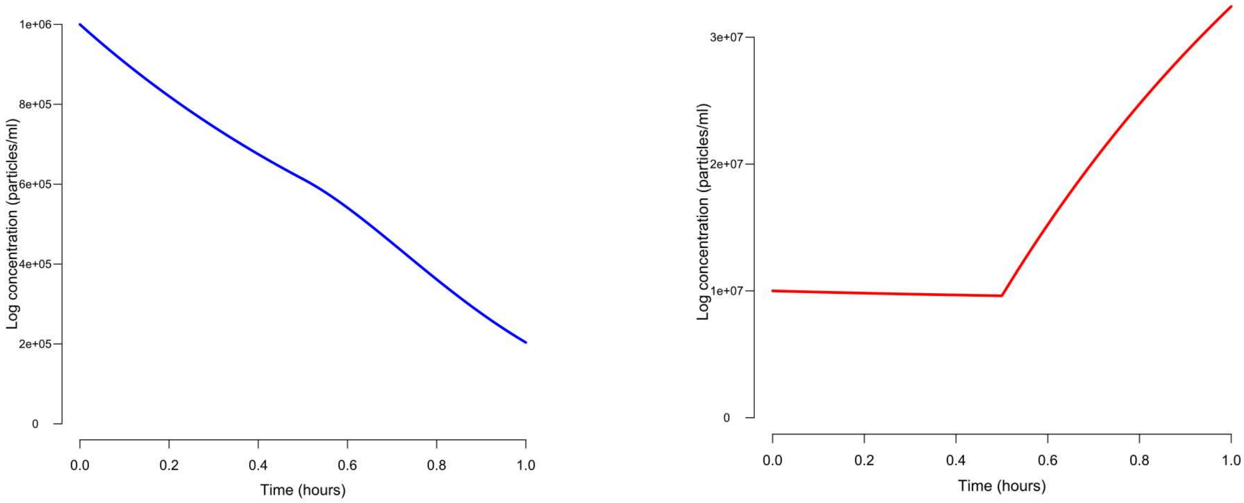

Figure S2. Investigating rate of bacterial eradication by simulation of bacteria and phage populations in a single well-mixed reactor over the course of one hour, as applies to the case study of fracking. The concentration of bacteria is shown in the left graph, while the concentration of phages is shown in the right graph. The simulation indicates that roughly 90% of all bacteria can be eradicated if 10^7 phages/ml are added to the reactor at the start of the time period. Parameters: infection rate $K_i = 10^{-7}$ ml/s, latency time $T = 0.5$ h, burst size $b = 60$.

References

Santos, S.B., Carvalho, C., Azeredo, J. and Ferreira, E.C. (2014) Population Dynamics of a Salmonella Lytic Phage and Its Host: Implications of the Host Bacterial Growth Rate in Modelling. PLoS ONE 9(7), e102507.
